# Delayed Gastric Emptying After Classical Pancreaticoduodenectomy Versus Pylorus-Preserving Pancreaticoduodenectomy With Billroth II Retrocolic Reconstruction in Patients With Cancer

**DOI:** 10.7759/cureus.97701

**Published:** 2025-11-24

**Authors:** Sergio Isidro Gamboa-Hoil, Ricardo Gamboa-Gutiérrez, Alejandro Medina-Campos

**Affiliations:** 1 Oncological Surgery, Instituto Mexicano del Seguro Social, Mérida, MEX; 2 Medical Research Unit, Instituto Mexicano del Seguro Social, Mérida, MEX

**Keywords:** billroth ii gastrojejunostomy, delayed gastric emptying, gastrojejunostomy, gastroparesis, pancreaticoduodenectomy, whipple procedure

## Abstract

Introduction

Delayed gastric emptying (DGE) is a common complication after pancreaticoduodenectomy (PD), associated with prolonged hospitalization and delayed initiation of adjuvant therapy. Surgical reconstruction technique may influence postoperative gastric motility. This single-center study evaluated the association between the type of PD (classical vs. pylorus-preserving PD (PPPD)) and the incidence of DGE in patients undergoing standardized retrocolic Billroth II reconstruction for malignant disease.

Materials and methods

We conducted a retrospective observational study of 15 patients who underwent PD for malignant disease between June 2021 and June 2022 at a tertiary oncology center. All patients underwent retrocolic Billroth II reconstruction. Clinically relevant DGE (grades B and C) was defined according to the International Study Group of Pancreatic Surgery criteria.

Results

DGE occurred in 46% (7/15) of patients overall. The incidence was significantly higher after classical PD (77.8%, 7/9) compared with no cases in the PPPD group (0/6; p = 0.007). Among patients with DGE, 28.6% (2/7) tolerated solid intake before postoperative week three, while 71.4% (5/7) did so after week three. Ninety-day mortality was 0% in both groups. At study closure, overall survival was 77.8% in the classical PD group and 100% in the PPPD group (p = 0.642), reported descriptively due to the limited sample size.

Conclusion

PPPD appeared to be associated with a lower frequency of clinically relevant DGE after standardized retrocolic Billroth II reconstruction. These preliminary findings suggest that meticulous, tension-free reconstruction may help optimize postoperative gastric motility. Larger studies are needed to validate these exploratory observations.

## Introduction

Delayed gastric emptying (DGE) was first described by Warshaw in 1985 and is defined as the inability to tolerate oral intake due to prolonged gastric stasis, with an incidence reported between 35% and 61% [1-3]. The pathophysiology is multifactorial, involving hormonal, neuromuscular, and surgical factors. Peptides such as cholecystokinin (CCK) and glucagon-like peptide-1 (GLP-1) delay gastric transit by increasing pyloric resistance, whereas motilin and ghrelin enhance gastric motility [4,5]. Reduced digestive enzyme activity may also contribute [6]. Beyond its multifactorial pathophysiology, DGE has important clinical implications as it directly affects the patient’s ability to resume oral intake, often resulting in inadequate caloric consumption and progressive nutritional decline. This deterioration in nutritional status may subsequently delay postoperative recovery and the initiation of adjuvant chemotherapy, a key determinant of long-term oncologic outcomes in patients with malignant diseases.

From a surgical perspective, disruption of antral-pyloric coordination and vagal nerve injury have been proposed as mechanisms for early postoperative DGE, even when no mechanical obstruction is present [7]. Thus, DGE remains a significant and multifactorial complication after pancreaticoduodenectomy (PD), influenced not only by physiological factors but also by the reconstruction technique employed.

However, evidence comparing DGE between classical PD and pylorus-preserving PD (PPPD) with standardized retrocolic Billroth II reconstruction remains limited. The primary objective of this single-center study was to evaluate the association between the type of PD (classical PD vs. PPPD) and the incidence of DGE in patients undergoing standardized retrocolic Billroth II reconstruction for malignant disease.

## Materials and methods

Patient selection

This was an observational, retrospective, and analytical study. Between June 2021 and June 2022, a total of 15 patients underwent PD for a confirmed cancer diagnosis at the Oncology Hospital, Unidad Médica de Alta Especialidad Yucatán, part of the Mexican Social Security Institute (Instituto Mexicano del Seguro Social). All eligible patients were included in the study.

Ethical considerations

The study protocol was approved by the Institutional Review Board (IRB No. 3203; Registration R-2023-3203-003). All procedures followed the Declaration of Helsinki. Written informed consent was obtained from all participants.

Surgical technique

All procedures were performed under general anesthesia using a supratransumbilical incision. Depending on tumor extension, either a classical PD or a PPPD was carried out. Clinical characteristics of the cohort were recorded following tumor resection [8]. All surgeries were performed by the same board-certified surgical oncologist, assisted by a general surgeon and/or general surgery residents.

A retrocolic ascent of the jejunal loop was achieved by opening the mesocolon to the left of the middle colic artery. The sequence of anastomoses was standardized as follows: first, a hepaticojejunostomy was constructed 20 cm from the ascended jejunal loop; second, a pancreaticojejunostomy was performed 5 cm from the blind end of the jejunal loop, using an end-to-side configuration and a modification of the Blumgart technique [9]; and finally, a gastrojejunostomy was completed retrocolically, 40 cm distal to the hepaticojejunostomy, following either the classical PD or PPPD approach. The ascended jejunal loop was subsequently secured to the mesocolon to maintain stability and prevent tension on the anastomoses.

Surgical techniques: classical PD vs. PPPD

Classical PD

**Figure 1:**
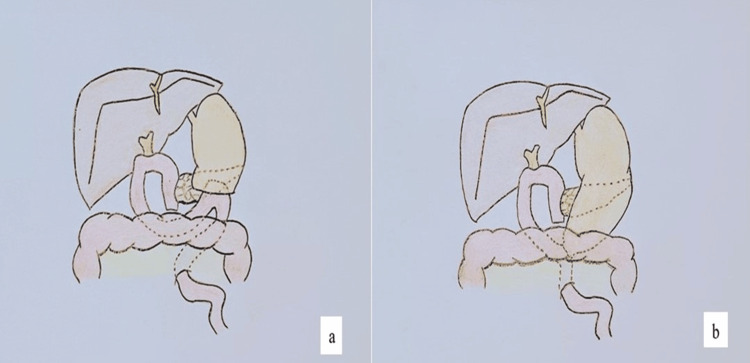
Pancreaticoduodenectomy Classical (a) or pylorus-preserving (b) pancreaticoduodenectomy, with Billroth II retrocolic reconstruction of the gastro or duodenojejunum anastomosis. Figure created by the author

A standard lymphadenectomy was performed, preserving the left gastric artery while dividing the right gastric artery, the right gastroepiploic artery, and the innervation of the distal stomach. Reconstruction was completed with an end-to-side gastrojejunostomy using the ascended jejunal loop. The anastomosis was created in two layers with the Connell-Mayo technique using 3-0 Vicryl and subsequently reinforced with 3-0 silk Lembert sutures (Figure 1a).

PPPD

Lymphadenectomy was performed with preservation of the left gastric artery, while the right gastric artery and the right gastroepiploic artery were divided. The pylorus was transected approximately 2 cm distal to the gastric outlet, and an end-to-side gastrojejunostomy was then constructed using the ascended jejunal loop. The anastomosis was performed in two layers with the Connell-Mayo technique using 3-0 Vicryl and reinforced with 3-0 silk Lembert sutures (Figure 1b).

Common technical details

In both approaches, two drains (19-20 Fr endopleural tubes) were placed: one positioned near the hepaticojejunostomy and the other near the pancreaticojejunostomy. Octreotide was not administered to any patient. Oral intake was initiated on postoperative day three.

Definition of outcomes

DGE was defined according to the criteria proposed by the International Study Group of Pancreatic Surgery (ISGPS) [10]. DGE was categorized into three grades (A, B, and C) based on the timing of nasogastric tube reinsertion, the persistence of gastrointestinal symptoms such as nausea or vomiting, and the patient’s ability to tolerate oral intake. Grade A was defined as the need for nasogastric decompression between postoperative days 4 and 7, or the inability to tolerate solid food by day 7, with recovery before day 14. Grade B corresponded to nasogastric tube use or reinsertion between days 8 and 14, or intolerance of solid food by day 14, with recovery before day 21. Grade C referred to nasogastric tube dependence or persistent intolerance of oral intake beyond day 14, with symptoms lasting past day 21. For the purposes of this study, only grades B and C, representing clinically relevant DGE, were included in the analysis.

Statistical analysis

Statistical analyses were performed using IBM SPSS Statistics for Windows, Version 25 (Released 2017; IBM Corp., Armonk, New York, United States). Continuous variables were first assessed for normality using the Kolmogorov-Smirnov test. Normally distributed variables were expressed as mean ± standard deviation and compared using the Student’s t-test. Non-normally distributed variables were expressed as median (range) and compared using the Mann-Whitney U test. Categorical variables were expressed as frequencies and percentages, and differences between groups were analyzed using the chi-square test or Fisher’s exact test, as appropriate. Relative risk (RR) with 95% confidence intervals (CIs) was calculated for factors associated with DGE. A p-value ≤ 0.05 was considered statistically significant.

## Results

Between June 2021 and June 2022, a total of 15 patients (5 men and 10 women) underwent pancreaticoduodenectomy for a cancer diagnosis at our institution. All procedures were performed by the same board-certified surgical oncologist, assisted by a general surgeon and/or general surgery residents. The mean age at diagnosis was 57.9 years (range: 31-75 years). The pancreatic head was the most common tumor site (53.3%), and adenocarcinoma was the predominant histological type (80%). The mean tumor size was 4.2 cm (range: 1-9.5 cm). The clinical and pathological characteristics stratified by type of reconstruction (classical PD vs. PPPD) are summarized in Table 1. 

**Table 1:** Clinical and pathological characteristics of the study population according to the procedure performed PD: pancreaticoduodenectomy *Fisher’s exact test; +Mann-Whitney U test; a p-value of ≤0.05 was considered statistically significant

Variable	Classical PD (n = 9)	Pylorus-preserving PD (n = 6)	p-value
Sex, n (%)			
Male	2 (22.2)	3 (50.0)	0.329*
Female	7 (77.8)	3 (50.0)	
Comorbidities, n (%)			
Diabetes mellitus	2 (22.2)	0 (0)	0.486*
Hypertension	6 (66.7)	4 (66.7)	1.000*
Heart disease	2 (22.2)	0 (0)	0.486*
Tumor location, n (%)			
Ampulla of Vater	0 (0)	4 (66.7)	
Pancreas	6 (66.7)	2 (33.3)	0.095*
Duodenum	3 (33.3)	0 (0)	
Histology, n (%)			
Adenocarcinoma	7 (77.8)	5 (83.3)	
Solid pseudopapillary neoplasm	2 (22.2)	0 (0)	0.865*
GIST	0 (0)	1 (16.7)	
Tumor size, cm, median (range)	5.0 (1.5-9.5)	2.5 (1.7-3.5)	0.456+
T stage, n (%)			
T1b	2 (22.2)	0 (0)	
T1c	4 (44.5)	0 (0)	
T2	0 (0)	4 (66.7)	0.264*
T3a	0 (0)	2 (33.3)	
T4	1 (11.1)	0 (0)	
Others	2 (22.2)	0 (0)	
N stage, n (%)			
N0	5 (55.6)	6 (100)	
N+	2 (22.2)	0 (0)	0.513*
Others	2 (22.2)	0 (0)	
M stage, n (%)			
M0	7 (77.8)	6 (100)	1.000*
M+	0 (0)	0 (0)	
Others	2 (22.2)	0 (0)	
Clinical stage, n (%)			
I	2 (22.2)	1 (16.7)	
IA	2 (22.2)	0 (0)	
IB	0 (0)	3 (50.0)	0.663*
IIA	0 (0)	2 (33.3)	
IIB	1 (11.2)	0 (0)	
III	2 (22.2)	0 (0)	
Others	2 (22.2)	0 (0)	
Margins, n (%)			
Negative	9 (100)	5 (83.3)	0.832*
Positive	0 (0)	1 (16.7)	
Neural invasion, n (%)			
Positive	3 (33.3)	0 (0)	0.356*
Negative	6 (66.7)	6 (100)	
Lymphovascular invasion, n (%)			
Positive	3 (33.3)	0 (0)	0.356*
Negative	6 (66.7)	6 (100)	
Histological grade, n (%)			
I	0 (0)	1 (16.7)	
II	4 (44.5)	5 (83.3)	0.574*
III	3 (33.3)	0 (0)	
Others	2 (22.2)	0 (0)	

Differences between classical PD and PPPD

The median intraoperative blood loss was 350 mL in the classical PD group and 700 mL in the PPPD group (Mann-Whitney U = 9.0; p = 0.036). A semi-soft pancreatic texture was observed in 80% of cases, with no significant difference between groups (p = 0.438). Pancreatic leakage occurred in one patient in the classical PD group (6.7%; p = 0.832). The median length of hospital stay for the entire cohort was eight days (Table 2). Adjuvant treatment was administered to 22% (2/9) of patients in the classical PD group and 100% (6/6) in the PPPD group (Fisher’s exact test, p = 0.007) (Table 3).

**Table 2:** Characteristics of the surgical procedure according to the procedure performed DGE: delayed gastric emptying; PD: pancreaticoduodenectomy * Fisher’s exact test; + Mann-Whitney U test; a p-value of ≤0.05 was considered statistically significant

Variable	Classical PD (n = 9)	Pylorus-preserving PD (n = 6)	p-value
DGE, n (%)			
Yes	7 (77.8)	0 (0)	0.007*
No	2 (22.2)	6 (100)	
DGE criteria, n (%)			
B	2 (28.6)	0 (0)	0.675*
C	5 (71.4)	0 (0)	
Readmissions due to intolerance to oral intake (median)	3	0	0.041+
Pancreatic duct size, mm, n (%)			
2	3 (33.3)	2 (33.3)	0.328+
3	6 (66.7)	1 (16.7)	
4	0 (0)	3 (50.0)	
Pancreatic texture, n (%)			
Soft	0 (0)	1 (16.7)	0.438*
Semi-soft	9 (100)	3 (50.0)	
Fibrous	0 (0)	2 (33.3)	
Intraoperative bleeding (categorical, n %)			
300	2 (22.2)	0 (0)	—
350	4 (44.5)	0 (0)	
400	0 (0)	3 (50.0)	
500	1 (11.1)	0 (0)	
800	2 (22.2)	0 (0)	
1000	0 (0)	3 (50.0)	
Intraoperative bleeding loss (mL, median)	350	700	0.036+
Intraoperative hemotransfusion (categorical, n %)			
0	4 (44.5)	1 (16.7)	—
1	3 (33.3)	2 (33.3)	
2	2 (22.2)	1 (16.7)	
3	0 (0)	2 (33.3)	
Units transfused, mean (SD)	1 (0.83)	1.5 (1.2)	0.181+
Reintervention, n (%)			
No	9 (100)	6 (100)	1.000*
Pancreatic leakage, n (%)			
Yes	1 (11.1)	0 (0)	0.832*
No	8 (88.9)	6 (100)	
Bile leakage, n (%)			
No	9 (100)	6 (100)	1.000*
Plasma albumin (g/dL, mean ± SD)	3.7 (0.41)	3.4 (1.1)	0.181+
90-day mortality, n (%)			
Alive	9 (100)	6 (100)	1.000*

**Table 3:** Distribution of adjuvant treatment according to the procedure performed PD: pancreaticoduodenectomy * Fisher’s exact test; a p-value of ≤0.05 was considered statistically significant

Variable	Classical PD (n = 9)	Pylorus-preserving PD (n = 6)	p-value
Adjuvant chemotherapy, n (%)			
Yes	2 (22.2)	6 (100)	0.007*
No	7 (77.8)	0 (0)	
Start of chemotherapy, weeks, n (%)			
4	0 (0)	4 (66.7)	0.414*
7	2 (100)	2 (33.3)	
Adjuvant radiotherapy, n (%)			
Yes	0 (0)	2 (33.3)	0.321*
No	9 (100)	4 (66.7)	
Start of radiotherapy, weeks, n (%)			
6	0 (0)	2 (100)	—

Delayed gastric emptying (DGE)

DGE was observed in 46% (7/15) of patients overall. It occurred in 77% (7/9) of patients in the classical PD group and in none of the PPPD group (Fisher’s exact test, p = 0.007). Regarding oral tolerance, 28% (2/7) of patients tolerated solid food intake before the end of the third postoperative week, while 71% (5/7) achieved tolerance after the third week (Table 2).

Postoperative morbidity and mortality

The 90-day mortality rate was 0% in both groups (Table 2). Tumor recurrence occurred in 22% (2/9) of patients in the classical PD group and 33% (2/6) in the PPPD group (p = 0.905). At study closure, overall survival was 77% (7/9) in the classical PD group and 100% (6/6) in the PPPD group (p = 0.642) (Table 4). Survival outcomes are reported descriptively to illustrate postoperative follow-up, acknowledging the limited sample size of the cohort.

**Table 4:** Recurrence and survival according to the procedure performed PD: pancreaticoduodenectomy * Fisher’s exact test; a p-value of ≤0.05 was considered statistically significant

Variable	Classical PD (n = 9)	Pylorus-preserving PD (n = 6)	p-value
Recurrence, n (%)			
Yes	2 (22.2)	2 (33.3)	0.905*
No	7 (77.8)	4 (66.7)	
Recurrence site, n (%)			
Local	0 (0)	2 (100)	0.317*
Local + liver + lung	2 (100)	0 (0)	
Time to recurrence, months, n (%)			
6	0 (0)	2 (100)	0.317*
7	2 (100)	0 (0)	
Overall survival at study closure, n (%)			
Alive	7 (77.8)	6 (100)	0.642*
Deceased	2 (22.2)	0 (0)	

Factors associated with DGE

Female sex was associated with a higher observed frequency of DGE (RR: 3.3; 95% CI: 1.2-8.5; p = 0.026). In contrast, undergoing PPPD showed a protective association (RR: 0.22; 95% CI: 0.06-0.75; p = 0.007) (Table 5).

**Table 5:** Factors associated with delayed gastric emptying DGE: delayed gastric emptying; RR: relative risk; CI: confidence interval * Fisher’s exact test; a p-value of ≤0.05 was considered statistically significant

Variable	DGE, n (%)	Relative risk (95% CI)	p-value
Female sex	7/7 (100)	3.3 (1.2-8.5)	0.026*
Pylorus-preserving PD	0/6 (0)	0.22 (0.06-0.75)	0.007*

## Discussion

Only patients with malignant pathology were included in this study. The mean age across both groups (classical PD and PPPD) was 57.9 years, which is consistent with values reported in the literature [2,11-16]. Adenocarcinoma was the most frequent histologic type (80%), and the pancreatic head was the most common tumor location (53.3%). The mean tumor size was 4.2 cm, comparable to previously published series [2,11-16].

Pancreatic leakage occurred in one patient in the classical PD group (6.7%; p = 0.832), a frequency lower than that described by Di Carlo et al., who reported incidences of up to 13% [12]. Our findings also differ from those of Busquets et al., who documented pancreatic leakage in 7% of patients undergoing classical PD and in 19% of those treated with PPPD [14].

In this single-surgeon cohort, DGE was observed in 46% (7/15) of patients, with a significantly higher incidence in the classical PD group (77%) compared to no cases in the PPPD group (Fisher’s exact test, p = 0.007). The overall incidence in our series was higher than that reported by Martignoni et al., who described a rate of 35% [2]. When analyzed by subgroup, the incidence of DGE after classical PD (77%) exceeded international reports, which range between 15% and 50%. Conversely, the absence of DGE in the PPPD group contrasts with published rates ranging from 12% to 62% [11-14,17].

With respect to severity, DGE type B was documented in 29% (2/9) of patients and type C in 71% (5/9), whereas Busquets et al. reported lower rates of type B (20%) and type C (35%) [14]. These findings differ from prior series, suggesting that, in addition to patient-related and physiological factors [4-7], the specific technique of the gastrojejunostomy or duodenojejunostomy may play a critical role in influencing postoperative gastric motility [18-20].

Several reconstruction techniques have been described for PD. In our series, we employed a retrocolic Billroth II reconstruction, a method previously associated with lower rates of DGE compared to Roux-en-Y or Billroth I anastomoses [21,22]. None of our patients underwent retromesenteric jejunal ascent, a technique linked to higher rates of DGE (p = 0.017) [23]. Although some studies suggest that antecolic routing may improve outcomes [24,25], findings remain inconsistent, with other reports demonstrating no significant differences [3]. The absence of DGE in the PPPD group (RR: 0.22; 95% CI: 0.06-0.75; p = 0.007), despite the retrocolic approach, suggests that factors such as standardized technique, tension-free anastomoses, and proper jejunal limb orientation may be more critical determinants of gastric motility than the reconstruction route itself. These observations are consistent with prior evidence underscoring the importance of meticulous surgical technique and pyloric preservation in minimizing postoperative gastric stasis [18,21,22,24,26,27].

In our study, female sex (RR: 3.3; 95% CI: 1.2-8.5; p = 0.026) was identified as a risk factor for DGE; however, previous studies have not demonstrated a consistent association between gender and DGE. This observation may reflect variability in case selection and the limited sample size, as prior reports have noted inconsistent or inconclusive results regarding gender as a determinant of DGE [23,27,28].

Our findings support the standardization of PPPD with meticulous reconstruction as a strategy to minimize DGE [11-14]. Given the significant impact of DGE on recovery, nutritional status, and initiation of adjuvant therapy, surgical planning should incorporate reconstruction techniques proven to reduce gastric stasis [18,21,22,24,25,27,29]. Despite technical advances, DGE remains a clinically relevant complication of PD, underscoring the importance of surgical expertise in optimizing gastric motility, preserving intestinal function, and minimizing morbidity in the postoperative setting [27,29].

Moreover, while postoperative biochemical and inflammatory factors have been implicated in the pathophysiology of DGE, these variables were beyond the scope of the present study, which primarily focused on the influence of surgical reconstruction technique. Nonetheless, acknowledging these mechanisms is important, as future research integrating both surgical and biochemical parameters may provide a more comprehensive understanding of the multifactorial nature of DGE and support the development of targeted preventive strategies.

Limitations of the study

This study has several inherent limitations. First, the sample size is small and limits the statistical power of the analyses, which may reduce the ability to detect subtle differences between groups. Second, the retrospective, nonrandomized design and the fact that all operations were performed by a single surgeon introduce potential selection bias and restrict external validity. Third, unmeasured perioperative or institutional factors may act as confounders that were not fully accounted for. For these reasons, the findings should be interpreted as exploratory and hypothesis-generating rather than definitive or generalizable. Larger multicenter studies are needed to validate these preliminary observations.

## Conclusions

PPPD appeared to be associated with a lower frequency of DGE in patients undergoing standardized Billroth II retrocolic reconstruction for malignancy. These preliminary findings suggest that surgical factors such as meticulous technique, tension-free anastomoses, and proper jejunal limb orientation may play an important role in postoperative gastric motility. Larger multicenter studies are needed to validate these exploratory results.
